# Home-Based Multidisciplinary Rehabilitation following Hip Fracture Surgery: What Is the Evidence?

**DOI:** 10.1155/2013/875968

**Published:** 2013-12-17

**Authors:** Kathleen Donohue, Richelle Hoevenaars, Jocelyn McEachern, Erica Zeman, Saurabh Mehta

**Affiliations:** ^1^School of Rehabilitation Science, McMaster University, Hamilton, ON, Canada L8S 1C7; ^2^School of Physical Therapy, Marshall University, 2847 5th Avenue, Huntington, WV 25705, USA

## Abstract

*Objective*. To determine the effects of multidisciplinary home rehabilitation (MHR) on functional and quality of life (QOL) outcomes following hip fracture surgery. *Methods*. Systematic review methodology suggested by Cochrane Collboration was adopted. Reviewers independently searched the literature, selected the studies, extracted data, and performed critical appraisal of studies. Summary of the results of included studies was provided. *Results*. Five studies were included. Over the short-term, functional status and lower extremity strength were better in the MHR group compared to the no treatment group (NT). Over the long-term, the MHR group showed greater improvements in balance confidence, functional status, and lower extremity muscle strength compared to NT group, whereas the effect on QOL and mobility was inconsistent across the studies. Several methodological issues related to study design were noted across the studies. *Conclusion*. The MHR was found to be more effective compared to the NT in improving functional status and lower extremity strength in patients with hip fracture surgery. Results of this review do not make a strong case for MHR due to high risk of bias in the included studies. Further research is required to accurately characterize the types of disciplines involved in MHR and frequency and dosage of intervention.

## 1. Introduction

Hip fracture is common across all age groups but is more common in older adults. Most hip fractures are treated surgically [1]. Hip fractures place tremendous burden on health care systems [2, 3]. Individuals with hip fractures have increased mortality, long-term disability, and functional dependence since most older adults do not attain pre-injury functional levels [4]. Moreover, impairment in quality of life (QOL), psychological and social domains, and fall related efficacy are also reported following a hip fracture [5, 6]. Specific interventions have been designed to reduce the impact of hip fracture on these domains. The components of an intervention depend on targeted outcome.

Individuals with hip fracture consider increase in mobility and functions to be the preferred outcomes when asked about their recovery expectations following hip fracture [7]. Postsurgical rehabilitation programs aim to reduce disability and improve mobility, functions, balance, strength and QOL following hip fracture [8]. They are implemented in variety of settings such as inpatient, outpatient, or home-based rehabilitation. Posthip fracture rehabilitation may involve multidisciplinary care, which includes services provided by multiple health disciplines such as physiotherapy (PT), occupational therapy (OT), nursing, social work, and dietetics under the supervision of a geriatrician or rehabilitation physician. Previous systematic reviews have indicated that multidisciplinary rehabilitation leads to superior outcomes in individuals following hip fracture surgery [8–10].

While there is evidence to believe that multidisciplinary rehabilitation is effective in individuals following hip fracture surgery, whether the recovery patterns differ depending on the setting (inpatient, outpatient, or home) in which it is provided is yet to be determined. Literature thus far has largely focused on examining the benefits of multidisciplinary rehabilitation rather than specifically investigating the influence of treatment setting in which such rehabilitation is delivered. Home-based rehabilitation presents a viable option compared to rehabilitation services delivered in inpatient or outpatient rehabilitation setting. Home-based rehabilitation typically occurs when a patient is discharged from an inpatient setting (acute care or subacute rehabilitation) and receives further rehabilitation at home to maintain continuum of care. Home-based rehabilitation programs facilitate early discharge from hospital, thereby reducing the financial burden associated with hip fractures. They are also safe and efficient in managing individuals following hip fractures [11]. Some of the previous studies have demonstrated that home-based rehabilitation improves physical function, health related quality of life (HRQOL), and balance confidence [11, 12]. However, these studies largely incorporate only PT services where the intervention is not multidisciplinary in nature. With distinct advantages of managing individuals in their home setting, it is important to examine the benefits of multidisciplinary home rehabilitation (MHR). In particular, a comprehensive review of literature to assess the effect of MHR on functions, mobility, balance, and HRQOL can facilitate understanding of appropriate discharge setting following hip fracture surgery.

The purpose of this study was to conduct a systematic review of randomized control trials (RCTs) to determine the effects of MHR on outcomes such as functional status, HRQOL, mobility, lower limb muscle strength, and balance following hip fracture surgery.

## 2. Methods

### 2.1. Criteria for including Studies

This review considered all parallel RCTs comparing MHR to inpatient, outpatient, or no treatment (NT) in patients following hip fracture surgery. We defined NT as the groups of patients who received conventional acute postsurgical care in hospital and was sent home with no further rehabilitation. In contrast, the inpatient and outpatient groups received multidisciplinary rehabilitation in inpatient and outpatient setting, respectively, following conventional acute care. The home-based group received multidisciplinary rehabilitation at home following their stay in acute care. Functional status, HRQOL, and balance confidence were the patient reported outcomes considered for this review. Physical mobility, functional status, lower limb strength, ambulation ability, and balance were the performance-based outcomes considered for the review.

Only RCTs in which one of the groups received MHR following hip fracture surgery were included. Prospective cohort and case-control studies were excluded from the review. Studies with patient groups other than those with hip fracture surgery, those with cost comparison as the primary outcome, or those with single-discipline home-based rehabilitation as the experimental group were also excluded. Trials looking at the effectiveness of multidisciplinary inpatient rehabilitation and those that examined the benefits of home-based physiotherapy alone were also excluded. Studies which examined the effects of MHR after elective hip surgery (such as total hip arthroplasty) were also excluded.

### 2.2. Search Techniques for Identifying Relevant Studies

A comprehensive literature search was performed on multiple databases to identify studies relevant to this review. Four student investigators (Kathleen Donohue, Richelle Hoevenaars, Jocelyn McEachern, and Erica Zeman) agreed upon the search terms to be used in each of the databases a priori. Two reviewers (Kathleen Donohue and Richelle Hoevenaars) performed the searches on the Ovid MEDLINE, EMBASE, PubMed, Cochrane Central Register of Controlled Trials, PEDro, CINHAL, and REHABDATA databases in isolation. ProceedingsFirst was searched to identify any relevant conferences and workshops abstracts suitable for this review. Relevant masters and/or doctoral theses were searched on ProQuest Dissertations and Thesis. The reference lists of relevant studies and systematic reviews were also searched and studies that appeared relevant in these lists were included.

### 2.3. Data Collection and Analysis

#### 2.3.1. Selection of Studies

The citations obtained from the search were screened by two independent reviewers (Jocelyn McEachern and Erica Zeman). A final list of eligible studies was prepared for full text review. Disagreements between the reviewers were resolved by discussion to reach a consensus. Agreement between the reviewers was assessed by examining unweighted kappa (*κ*).

#### 2.3.2. Data Extraction and Management

Data extraction was performed independently by two reviewers (Jocelyn McEachern and Erica Zeman). Both reviewers were in the final year of their entry-level physiotherapy program at McMaster University, Hamilton, Canada. A standardized data collection form was generated for this study. Descriptive data regarding number of participants, their age and sex, and the study setting were extracted. Methodology regarding generation of randomization sequence, allocation concealment, blinding, and completeness of outcome data were extracted to gauge the risk of bias [13]. Information related to the intervention such as types of rehabilitation, frequency of visits, and duration of treatments was also extracted.

#### 2.3.3. Assessment of Risk of Bias

An assessment of the risk of bias in the included studies was completed. Bias was defined as a systematic error in the truth of results or inferences [13]. A domain-based evaluation was used for the risk of bias assessment. The domains included were, random sequence generation, allocation concealment, blinding of outcome assessment and incomplete outcome data.

#### 2.3.4. Measurement of Treatment Effect

The outcomes were divided and examined as being short-term (the first four months) and long-term (12 months) in order to avoid the unit of analysis issues while performing analysis [14]. We had planned to examine standardized mean differences for all the outcomes (patient reported and performance-based) if the data across the included studies was deemed to be suitable for pooling. However, we chose to provide the summary of results and provide point estimates of treatment effects given that the meta-analysis was not feasible due to heterogeneity in the study design, participants, outcomes, interventions, and data reporting.

#### 2.3.5. Assessment of Reporting Biases

To determine any influence on the nature or direction of results, reporting biases were assessed [15]. A funnel plot to assess publication bias could not be plotted since only five studies were included in this review. We used comprehensive search including grey literature such as conference proceedings, dissertations, and master and doctoral theses to ensure that the possibility of the publication bias was minimized. Citation bias was limited as bibliographies of relevant systematic reviews were searched. Location and language biases were minimized by avoiding location and language limitations for the literature search.

#### 2.3.6. Subgroup Analysis and Investigation of Heterogeneity

Due to the nature and intensity of the multidisciplinary rehabilitation provided in different settings, we did not find it appropriate to combine the results and assess the treatment effect of MHR versus other comparison groups combined together. Rather, we opted to create subgroups and assess the treatment effect of the MHR (MHR versus institution-based rehabilitation and MHR versus NT).

Sensitivity Analysis was not required since no data imputations were done.

## 3. Results

**Figure 1:**
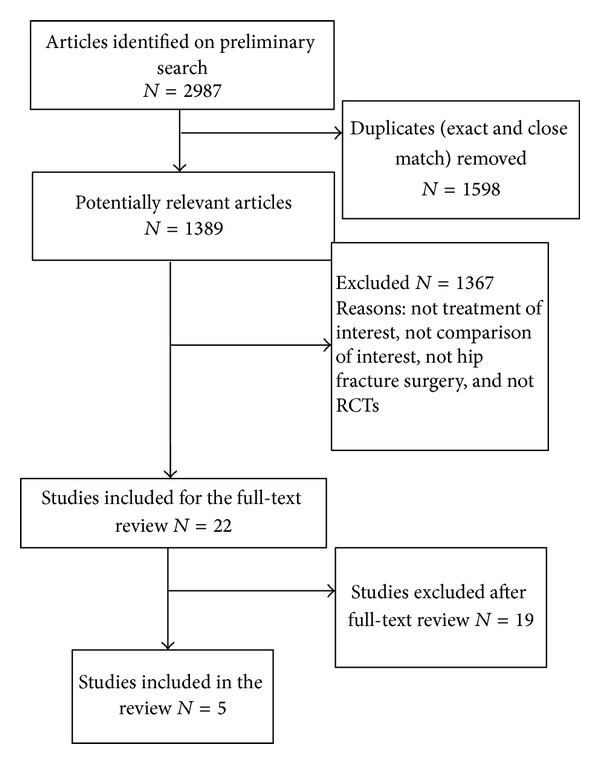
Flow Diagram.

The literature search strategies are outlined in Figure 1. The preliminary search identified 2987 citations. Twenty-two studies were selected for full text review. Five studies met the predefined inclusion/exclusion criteria and were chosen for this review. The process used to identify and select the studies used in this review is depicted in a flow diagram in Table 1. The raw agreement between two independent reviewers (Jocelyn McEachern & Erica Zeman) for inclusion of studies in this review was 86% with an unweighted *κ* of 0.73, which is considered acceptable [16].

### 3.1. Description of Included Studies

Of the five studies included, two were conducted in Sweden [17, 18], two in Australia [11, 19], and one in Hong Kong [20]. Comparator group was inpatient hospital-based setting in one [20] and NT in the other four studies [11, 17–19]. The treatment groups within all studies were discharged from inpatient hospital care to home where they received MHR. The number and disciplines that composed the multidisciplinary rehabilitation team varied between studies but all of them included physiotherapy (PT). The follow-up period varied between 1 month and 12 months between studies. A detailed description and characteristics of all included studies are expressed in Table 1.

### 3.2. Description of Excluded Studies

Nineteen studies were excluded after full text review. The reasons for exclusion were that the intervention was not provided by a multidisciplinary team (*n* = 11), participants had elective hip replacement surgery (*n* = 3), comparator group of interest was lacking (treatment and control group both had home-based intervention) (*n* = 2), treatment group did not have home-based intervention (*n* = 1), there was no RCT design (*n* = 1). A recent study conducted by Tseng et al. [21] was also excluded after careful review. The objective of their study was to assess the effects of interdisciplinary intervention on different trajectories of recovery (poor, moderate, and excellent) in individuals with hip fractures and not necessarily provide a direct comparison between the MHR group and the usual care group.

### 3.3. Risk of Bias in Included Studies

The agreement between reviewers for assessing the risk of bias was substantial, with an unweighted *κ* of 0.70 and a raw agreement of 84% [16].Table 2 summarizes risk of bias assessment for the included studies.

Despite the assumption that the original and follow-up articles would have identical protocols, the risk of bias in the included studies was assessed for Crotty et al. [11, 19] studies as well as the Zidén et al. [17, 18] with both the pairs reporting short-term and long-term results of their respective projects. This is because some of the aspects of the publication such as the blinding of outcomes assessors at different time points, incomplete outcome data reporting, and selective outcome reporting could be different for the same study for which the results were reported in two separate publications. While Crotty et al. [19] had very low risk of bias, their earlier publication [11] had risk of bias due to inadequate details of allocation concealment and incomplete data reporting. The overall risk of bias was low in one study [19], unclear in three studies [11, 17, 18], and high in one study [20].

### 3.4. Effects of Interventions

After comparing the results of outcomes reported either on the same scale or across different scales that measure the same construct, it was determined that pooling the results was not possible or appropriate. Therefore, a narrative summary of results was provided for short- and long-term time frames. The study by Kuisma [20] is being excluded from the following summary of results due to high risk of bias and lack of reporting of outcomes of interest.

#### 3.4.1. Patient Reported Balance Confidence

The effect of MHR on balance confidence over short-term was inconsistent across two studies [11, 17]. Crotty et al. [11] assessed balance confidence using the Activity Specific Balance Confidence (ABC) Scale and the FES. No significant differences were reported between the MHR group and the NT group on the ABC scale at 4 months (MHR median: 61.3, IQR: 45.5, 75.2; NT median: 53.3, IQR: 26.8, 74.6; *P* > 0.05); significant improvements in the FES scores were reported (MHR median: 90.5, IQR: 80.5, 98.0; NT median: 79.5, IQR: 40.0, 92.5; *P* < 0.05). Zidén et al. [17] measured patient reported balance confidence using the FES and found that the individuals in MHR group had increased balance confidence compared to those of the NT group at one-month followup (MHR mean (SD): 30.6 (19.3), NT mean (SD): 13.5 (20.9), and *P* = 0.0004).

Only Zidén et al. [18] measured patient reported balance confidence using the FES in the long-term (1 year after discharge). The FES scores indicated significantly higher balance confidence in MHR group compared to the NT group (MHR median (min-max): 128 (61–130); NT median (min-max): 102 (13–130); *P* < 0.001).

#### 3.4.2. Patient Reported Functional Status

The effect of MHR on functional status over short-term was also inconsistent across two studies [11, 17]. Crotty et al. [11] found no difference in functional status between the MHR and the NT groups at 4 months as measured by the London Handicap scores (MHR median: 0.70, IQR: 0.63,0.77; NT median: 0.65, IQR: 0.58, 0.73; *P* > 0.05). Zidén et al. [17] measured patient reported functional status using Frenchay's Activity (FAI). The results indicated that the scores on the FAI domestic activities subscale were significantly higher in the MHR group (MHR mean (SD): 9.0 (5.0); NT mean (SD): 6.4 (5.3); *P* = 0.0119). Similarly, the scores on the outdoor activities subscale were also significantly higher in the MHR group (MHR mean (SD): 5.7 (4.8); NT mean (SD): 2.7 (3.8); *P* = 0.0007). However, the scores on the leisure and work activity subscale did not differ between the groups at the one-month followup (MHR mean (SD): 3.4 (2.3); NT mean (SD): 2.6 (2.3); *P* = 0.1057).

#### 3.4.3. Patient Reported Functional Status (Long-Term)

Zidén et al. [18] evaluated functional status using the FAI in the long-term (1 year after discharge). Significantly higher scores in favour of the MHR group were found (*P* = 0.028) at 12 months (MHR median: (min-max), 27 (0–40); NT median: (min-max) 20 (0–42)).

#### 3.4.4. Patient Reported QOL (Short-Term)

Crotty et al. [11] evaluated QOL at 4 months using the London Handicap Scale and the SF-36 and found no significant differences between groups. (London Handicap Scale (MHR mean (95% CI): 0.70 (0.63–0.77), NT mean (95% CI): 0.65 (0.58–0.73); SF-36 on both the PCS component (NT mean (95% CI): 26.9 (10.2–42.0), MHR mean (95% CI): 38.3 (27.9–48.7), *P* > 0.05) and the MCS component (NT mean (95% CI): 42.8 (31.2–54.4); MHR mean (95% CI): 46.4 (36.2–56.6); *P* > 0.05).

Crotty et al. [19] and Zidén et al. [18] evaluated QOL over a longer term using SF-36. The effect of MHR on QOL was inconsistent across both studies. Crotty et al. [19] found no significant differences between groups on the SF-36 for both the PCS component (mean difference (95% CI): −2.3(−7.6–3.0), *P* = 0.386) and the MCS component (mean difference (95% CI): 0.7 (−3.8–5.3); *P* = 0.733). Zidén et al. [18] reported a significant improvement in the SF-36 domains of physical functioning (*P* = 0.001) and bodily pain (*P* = 0.042) in the MHR group at one-year followup.

#### 3.4.5. Mobility

The effect of MHR on mobility as assessed by the timed up and go (TUG) over a short-term period was inconsistent across two studies [11, 17]. Crotty et al. [11] found no significant difference in TUG scores at four months between the MHR and the NT groups (MHR median: 23.0, IQR: 15.3, 33.0; NT median: 28.0, IQR: 18.0, 42.5). In contrast, Zidén et al. [17] found significant differences in the TUG scores in favor of the MHR group compared to the NT group at one month (MRR mean (SD): 24.9 (15.4); NT mean (SD): 30.8 (6.0); *P* = 0.0139).

#### 3.4.6. Mobility (Long-Term)

Similar to the short-term followup, the effect of MHR on mobility as assessed by the TUG was also inconsistent across two studies [18, 19]. Crotty et al. [19] found that the median difference between the TUG scores of the MHR and the NT groups at 12 months was not significant (median difference (95% CI): −3.0 (−11.0 to 3.0); *P* = 0.314), while Zidén et al. [18] reported that TUG scores in the MHR group were significantly better than NT group (*P* = 0.005) at 12 months.

#### 3.4.7. Lower Extremity Functional Strength (Short-Term and Long-Term)

Zidén et al. [17] found that lower extremity strength assessed by the Sit-to-Stand (STS) test was significantly better the MHR group (*P* = 0.011) in comparison to the NT group in. This improvement was sustained over the 12-month followup as well and the MHR group continued to show significantly better lower extremity strength compared to the NT group (*P* < 0.001) [18].

#### 3.4.8. Balance (Short-Term)

The balance was assessed at four-months followup using the Berg Balance Scale in only one study [11]. The result indicated that the MHR group had higher scores indicating greater improvement compared to the NT group at four months; however the difference was not significant (MHR median: 43.5, IQR: 34.3, 52.5; NT median: 37.5 IQR: 26.3, 45.3; *P* > 0.05).

#### 3.4.9. Adverse Events

No adverse effects of the MHR group were reported in any of the studies reviewed [11, 17–19].

## 4. Discussion

In the short-term, the MHR group showed greater improvements in functional status and lower extremity strength but no difference in balance and HRQOL compared to the NT group. The effect of MHR on balance confidence and mobility was inconsistent across the studies, and therefore no inferences can be made whether MHR is effective in improving these outcomes.

In the long-term, the MHR group showed greater improvements in balance confidence, functional status, and lower extremity muscle strength compared to NT group. The effect of MHR on HRQOL and mobility was inconsistent across the studies.

The studies evaluated within this review suggest a trend towards positive outcomes from MHR program of care compared to no treatment following hip fracture surgery. Due to the low number of studies that fit the criteria for this review, robust conclusions cannot be made. Meta-analysis allows combining data across many studies and enables to derive “pooled” effect. However, the lack of consistency across outcome measures used in the studies limited our ability to perform meta-analysis. Furthermore, applying the evidence included in this review will be difficult, as the studies did not provide clear descriptions of the interventions administered. Though we realize that it may be unrealistic to provide explicit protocols, as treatment in a MHR setting will naturally vary between geographical areas, at least a detailed information regarding the specific roles of health care providers involved in the MHR plan would have been useful.

Although we only included the studies that reflect the best evidence into this review by deciding, a priori, to limit our search to RCTs alone, the quality of the included studies was limited both by risk of bias and methodological issues. Some of the key methodological issues observed across the included studies such as unclear in reporting methods of random sequence generation, lack of blinding of outcome assessment, and incomplete outcome data reporting can highly influence the results. Furthermore, many of these studies did not adequately describe the MHR provided, which further limits the generalizability of the results. Therefore, the results of this review do not allow us to make robust conclusions regarding the usefulness of the MHR following hip fracture surgery in comparison to no treatment.

A comprehensive search strategy performed in duplication, an assessment of agreement for selection of studies, and risk of bias assessment for included studies all contributed to the strength of this review. Criteria for inclusion/exclusion of studies were determined a priori and two reviewers extracted data from the studies using a previously developed and standardized data extraction form.

The comprehensive search strategies utilized in this review helped to minimize potential bias in the review process. Having two reviewers independently performing literature searche on a multitude of databases helped to ensure that all relevant articles were located and included in this review. However, outside of the reviewer's control, publication bias may still have influenced our outcomes. Although we set out to locate all studies on the topic of interest, it is possible that unpublished studies exist that may or may not have changed the results of our review.

In the study selection and data collection processes, we again minimized bias by performing these processes in duplicate. In the end there was some disagreement amongst reviewers as to which studies to include in the review and on the quality of included studies. Although this was resolved through discussion amongst all reviewers, it is possible that the subjectivity of this process may have introduced some bias into the review.

Lastly, studies did not clearly report details of the treatment delivered to the MHR group. Therefore, it is possible that the treatments varied in terms of content, duration, and frequency among the MHR groups across studies. We did not account for this variation when determining the treatment effect of MHR after hip fracture surgery. This may have introduced bias into the review, however we determined that the variation amongst home-based treatments could be overlooked as this is likely a realistic representation of the approach taken to home-based treatments in the community.

This systematic review expands on our systematic review by Mehta and Roy [22], which concluded that home-based physiotherapy was effective in improving patient reported health related QOL following hip fracture surgery. The outcomes investigated in this review were different from those of other similar reviews that assessed outcomes such as mortality or admission to long-term care homes [23, 24]. Moreover, the objective of our review was different in that it compared multidisciplinary rehabilitation provided in home versus that provided in institution as well as no treatment following discharge from acute hospital setting. However, our results agree with those of Handoll et al. [8] who suggested that multidisciplinary rehabilitation may have some benefit to patients following hip fracture but emphasized the need for high quality RCT to comprehensively examine the benefits of multidisciplinary rehabilitation.

## 5. Conclusions

Our findings show a trend to support MHR compared to NT following hip fracture surgery. In all instances, MHR was found to be at least as effective as, if not more than, NT. However, no conclusive directives can be made to adopt MHR following hip fracture surgery due to risk of bias across included studies and insufficient details regarding the components of the MHR in the studies. The poor methodological quality of studies included in this review underscores the need to conduct studies with superior methodological quality to investigate the effects of MHR in comparison with NT or institution-based care (inpatient or outpatient) in individuals with hip fracture surgery. Furthermore, future studies can accurately characterize the types of disciplines involved in MHR and frequency and dosage of intervention. Moreover, researchers involved in assessing the outcomes of hip fracture surgery can develop a core set of measures for different constructs (e.g. functional status, balance) relevant to hip fracture population. This will facilitate use of similar measures across future studies allowing meta-analysis and assessment of pooled effect of an intervention in individuals with hip fracture. Lastly, a cost analysis comparing NT or institution-based rehabilitation to home-based is necessary to ascertain the cost benefits of each mode of treatment.

## Figures and Tables

**Table 1:** Characteristics of the Included Studies.

Study	Method	Participants	Intervention	Outcomes	Results
Kuisma [20]	Parallel group randomized controlled equivalence trial; MHR versus inpatient rehabilitation	(i) *N* = 81 (40 in the MHR group and 41 in the inpatient group); (ii) Location—Hong Kong; (iii) Inclusion/exclusion criteria specified—partially; (iv) Age (y)—75 ± 8.3 for both groups; (v) Sex—49 females (60%) and 32 males (40%); (vi) Duration since hip fracture or surgery—not reported; (vii) Comorbidities reported—no	**MHR group** (i) No description provided for the nature of exercises. (ii) Average of 4.6 ± 2.2 PT visits and average of 1.5 ± 0.6 community nurse visits. **Inpatient group** (i) No description provided for the nature of exercises. (ii) Average stay in rehabilitation hospital was 36.2 ± 14.6 days with daily PT visits.	**PRO** None used **PBO** *Ambulatory capacity* Measured across five categories: community ambulatory, house-hold ambulatory, walking on flat surface, transfer from bed to chair, bed to chair bound. (scoring: 4 = independent without aids, 3 = independent with aids, 2 = able to walk with minimum assistance/supervision, 1 = able to walk with maximum assistance, 0 = unable to walk)	(i) Lost to followup was 9 in the MHR group and 16 in the inpatient group. Causes of attrition were not provided. (ii) Adverse events not reported. (iii) Community ambulatory ability was better in the MHR group. Flat-level ambulatory ability was similar in both the groups.

Crotty et al. [11]	Parallel group randomized controlled equivalence trial; MHR versus NT	(i) *N* = 66 (34 in the MHR group and 32 in the NT group; (ii) Location—Australia; (iii) Inclusion/exclusion criteria specified—yes; (iv) Age (y)—median 81.6 (IQR: 78.2, 85.4) in the MHR group and median 83.5 (IQR: 76.6–85.5) in the NT group; (v) Sex—21 females (62%) in the MHR group and 24 females (75%) in NT group; (vi) Average duration since surgery—not reported; (vii) Comorbidities reported—no	**MHR group** (i) Home visit to organize modifications and install equipment prior to discharge. (ii) Visited by therapists (team coordinator, PT, OT, SLP, SW, and therapy aid) within 48 hours of discharge. (iii) Frequency of therapy was tailored for individualized needs. Structured practice sessions encouraged between visits. (iv) Services such as podiatry, nursing care and assistance with light domestic tasks were provided as required. **NT group**: Received routine hospital care: inpatient services and the development of care pathways and discharge planning.	**PRO** *Balance Confidence* ABC Scale *Functional Status* (i) MBI (ii) LHS *HRQOL* (i) LHS (ii) SF-36 (PCS and MCS) **PBO** *Balance* BBS *Physical mobility* TUG *Falls* Frequency of falls and falls that requirehospitalization	(i) Attrition or causes of attrition were not specified. (ii) Adverse events were not reported. (iii) Patients in the MHR group did not show improvement in physical health but reported improvement in ADL. (iv) MHR group also had greater confidence in avoiding falls at four months.

Zid$\overset{´}{\text{e}}$n et al. [17]	Parallel group randomized controlled equivalence trial; MHR versus NT	(i) *N* = 102 (48 in MHR group and 54 in NT group); (ii) Location—Sweden; (iii) Inclusion/exclusion criteria specified—yes (iv) Age (y)—81.2 ± 5.9 in MHR group and 82.5 ± 7.6 in NT group; (v) Sex—29 females (60.4%) in the MHR group and 42 females (77.8%) in the NT group; (vi) Comorbidities reported—yes.	**MHR group** (i) Three weeks of rehabilitation that included PT to encourage self-efficacy and OT to encourage activity and independence in ADL. (ii) Participants received a mean of 4.9 (±0.4) multiprofessional home visits; 2.4 PT (±1.7) and 1.6 OT (±1.7) visits. (iii) Every fourth patient received a visit by a nurse. **NT group** No additional treatment after being discharged from hospital.	**PRO** *Balance Confidence* Swedish version of the FES (S) *Functional Status* (i) FIM motor scale (ii) Instrumental Activity Measure (measures degree of independence in eight advanced activities) (iii) Frenchay's Activity Index (determines the frequency of performing social and complex daily activities) **PBO** *Physical Mobility* TUG *Lower Extremity Strength* STS test	(i) Lost to follow up was 6 in the MHR group; causes of attrition were provided. (ii) No adverse events were reported (iii) The MHR group showed significant improvements on the FES, TUG, STS, FIM, Instrumental Activity Measure, and the Frenchay's Activity Index one month after discharge compared to the NT group.

Zid$\overset{´}{\text{e}}$n et al. [18]	Parallel group randomized controlled equivalence trial; MHR versus NT	(i) *N* = 102 (48 in the MHR group, 54 in the NT group); (ii) Location—Sweden; (iii) Inclusion/exclusion criteria specified—partially; (iv) Age (y)—81.2 ± 5.9 in MHR group and 82.5 ± 7.6 in NT group; (v) Sex—29 females (60.4%) in the MHR group and 42 females (77.8%) females in the NT group; (vi) Average duration since surgery—not reported; (vii) Comorbidities reported—yes	**MHR group** (i) Three weeks of rehabilitation that included PT to encourage self-efficacy and OT to encourage activity and independence in ADL. (ii) Median of visits 4.5 (with median of 3 PT visits and 1.5 OT visits, 11 patients were visited by a nurse). **NT group** No additional treatment after being discharged from hospital.	**PRO** *Balance Confidence* FES(S) *Functional Status* (i) FIM (ii) Instrumental Activity Measure (iii) Frenchay's Activity Index *HRQOL* SF-36 *Mood* Centre for Epidemiological Studies Depression Scale (screening instrument to measure depressive symptoms) **PBO** *Physical Mobility* TUG *Lower Extremity Strength* STS	(i) One participant in the MHR group and 3 in the NT group were lost at 6-month followup; additional 3 participants in the MHR group and 4 participants in the NT group were lost to followup at 1-year (reasons for attrition were provided). (ii) The MHR group had greater balance confidence and physical function than the NT group over 1 year period after hip fracture. (iii) One year after discharge 29% of the people in the MHR group considered themselves fully recovered, compared to only 9% in the NT group.

Crotty et al. [19]	Parallel group randomized controlled equivalence trial; MHR versus NT	(i) *N* = 56 (not clear about how many in each group); (ii) Location—Australia; (iii) Inclusion/exclusion criteria specified—yes (iv) Age (y) —mean of 81.8 ± 7.2 for both groups combined; (v) Sex—41 (73%) females for both groups combined; (vi) Average duration since surgery—not reported; (vii) Comorbidities reported—no.	**MHR group** (i) Home visits by PT, OT, SLP, SW, and therapy aides. (ii) Services such as podiatry, nursing care, and assistance with light domestic tasks were provided as required. **NT group**: Received routine hospital care, inpatient services, development of care pathways, and discharge planning.	**PRO** *Functional Status* Modified Barthel Index *HRQOL* SF-36 (MCS & PCS) **PBO** *Physical Mobility* TUG	(i) Ten patients unavailable for 12-month followup (reasons for attrition were provided). (ii) At 12 months, there were no differences between the groups for scores on the MBI, TUG, or SF-36 outcomes (PCS and MCS).

MHR: multidisciplinary home rehabilitation; IQR: interquartile range; NT: no treatment; PT: physiotherapist; OT: occupational therapist; SLP: speech language pathologist; SW: social worker; ADL: activities of daily living; PRO: patient-reported outcome; PBO: performance-based outcomes; ABC: activity-specific balance confidence scale; MBI: Modified Barthel Index; LHS: London Handicap Scale; SF-36: Short-Form-36; PCS: physical component summary; MCS: mental component summary; BBS: Berg balance scale; TUG: timed up and go test; FES: falls efficacy scale; FIM: functional independent measure; STS: sit-to-stand.

**Table 2:** Risk of bias assessment of included studies (Yes—No risk of bias, No—clear risk of bias, Unclear—indicates that risk of bias is unclear).

	Random sequence generation (selection bias)	Allocation concealment (selection bias)	Blinding of outcome assessment (detection bias)	Incomplete outcome data (attrition bias)	Selective reporting (reporting bias)
Crotty et al. 2002 [11]	Yes	Unclear	Yes	Unclear	Yes
Crotty et al. 2003 [19]	Yes	Yes	Yes	Yes	Yes
Kuisma 2002 [20]	Unclear	Yes	Yes	No	No
Zid$\overset{´}{\text{e}}$n et al. 2008 [17]	Unclear	Yes	No	Yes	Yes
Zid$\overset{´}{\text{e}}$n et al. 2010 [18]	Unclear	Yes	No	Yes	Yes

## References

[1] Handoll HH, Sherrington C, Mak JC (2011). Interventions for improving mobility after hip fracture surgery in adults. *Cochrane Database of Systematic Reviews*.

[2] Kaffashian S, Raina P, Oremus M (2011). The burden of osteoporotic fractures beyond acute care: the Canadian Multicentre Osteoporosis Study (CaMos). *Age and Ageing*.

[3] Leslie WD, Metge CJ, Azimaee M (2011). Direct costs of fractures in Canada and trends 1996–2006: a population-based cost-of-illness analysis. *Journal of Bone and Mineral Research*.

[4] Crotty M, Unroe K, Cameron ID, Miller M, Ramirez G, Couzner L (2010). Rehabilitation interventions for improving physical and psychosocial functioning after hip fracture in older people. *Cochrane Database of Systematic Reviews*.

[5] Chiu M-H, Hwang H-F, Lee H-D, Chien D-K, Chen C-Y, Lin M-R (2012). Effect of fracture type on health-related quality of life among older women in Taiwan. *Archives of Physical Medicine and Rehabilitation*.

[6] Visschedijk J, van BR, Hertogh C, Achterberg W (2013). Fear of falling in patients with hip fractures: prevalence and related psychological factors. *Journal of the American Medical Directors Association*.

[7] Milte R, Ratcliffe J, Miller M, Whitehead C, Cameron ID, Crotty M (2013). What are frail older people prepared to endure to achieve improved mobility following hip fracture? A Discrete Choice Experiment. *Journal of Rehabilitation Medicine*.

[8] Handoll HH, Cameron ID, Mak JC, Finnegan TP (2009). Multidisciplinary rehabilitation for older people with hip fractures. *Cochrane Database of Systematic Reviews*.

[9] Momsen AM, Rasmussen JO, Nielsen CV, Iversen MD, Lund H (2012). Multidisciplinary team care in rehabilitation: an overview of reviews. *Journal of Rehabilitation Medicine*.

[10] Cameron ID (2005). Coordinated multidisciplinary rehabilitation after hip fracture. *Disability and Rehabilitation*.

[11] Crotty M, Whitehead CH, Gray S, Finucane PM (2002). Early discharge and home rehabilitation after hip fracture achieves functional improvements: a randomized controlled trial. *Clinical Rehabilitation*.

[12] Tsauo J-Y, Leu W-S, Chen Y-T, Yang R-S (2005). Effects on function and quality of life of postoperative home-based physical therapy for patients with hip fracture. *Archives of Physical Medicine and Rehabilitation*.

[13] Higgins JPT, Altman DG, Higgins JPT, Green S (2008). Assessing risk of bias in included studies. *Cochrane Handbook for Systematic Reviews of Interventions Version 5.0.1*.

[14] Deeks JJ, Higgins JPT, Altman DG, Higgins JPT, Green S (2008). Analysing data and undertaking meta-analyses. *Cochrane Handbook for Systematic Reviews of Interventions Version 5.0.1*.

[15] Sterne JAC, Egger M, Moher D, Higgins JPT, Green S (2008). Addressing reporting biases. *Cochrane Handbook for Systematic Reviews of Intervention. Version 5.0.1*.

[16] Landis JR, Koch GG (1977). The measurement of observer agreement for categorical data. *Biometrics*.

[17] Zidén L, Frandin K, Kreuter M (2008). Home rehabilitation after hip fracture. A randomized controlled study on balance confidence, physical function and everyday activities. *Clinical Rehabilitation*.

[18] Zidén L, Kreuter M, Fränndin K (2010). Long-term effects of home rehabilitation after hip fracture—1-year follow-up of functioning, balance confidence, and health-related quality of life in elderly people. *Disability and Rehabilitation*.

[19] Crotty M, Whitehead C, Miller M, Gray S (2003). Patient and caregiver outcomes 12 months after home-based therapy for hip fracture: a randomized controlled trial. *Archives of Physical Medicine and Rehabilitation*.

[20] Kuisma R (2002). A randomized, controlled comparison of home versus institutional rehabilitation of patients with hip fracture. *Clinical Rehabilitation*.

[21] Tseng MY, Shyu YI, Liang J (2012). Functional recovery of older hip-fracture patients after interdisciplinary intervention follows three distinct trajectories. *Gerontologist*.

[22] Mehta SP, Roy J-S (2011). Systematic review of home physiotherapy after hip fracture surgery. *Journal of Rehabilitation Medicine*.

[23] Halbert J, Crotty M, Whitehead C (2007). Multi-disciplinary rehabilitation after hip fracture is associated with improved outcome: a systematic review. *Journal of Rehabilitation Medicine*.

[24] Bachmann S, Finger C, Huss A, Egger M, Stuck AE, Clough-Gorr KM (2010). Inpatient rehabilitation specifically designed for geriatric patients: systematic review and meta-analysis of randomised controlled trials. *British Medical Journal*.

